# Author Correction: Characterization of spike glycoprotein of SARS-CoV-2 on virus entry and its immune cross-reactivity with SARS-CoV

**DOI:** 10.1038/s41467-021-22614-1

**Published:** 2021-04-01

**Authors:** Xiuyuan Ou, Yan Liu, Xiaobo Lei, Pei Li, Dan Mi, Lili Ren, Li Guo, Ruixuan Guo, Ting Chen, Jiaxin Hu, Zichun Xiang, Zhixia Mu, Xing Chen, Jieyong Chen, Keping Hu, Qi Jin, Jianwei Wang, Zhaohui Qian

**Affiliations:** 1 NHC Key laboratory of Systems Biology of Pathogens, Institute of Pathogen Biology, Chinese Academy of Medical Sciences and Peking Union Medical College, Beijing, China; 2 Institute of Medicinal Plant Development (IMPLAD), Chinese Academy of Medical Sciences (CAMS) and Peking Union Medical Collage (PUMC), Beijing, China; 3 Hengshui Third People’s Hospital, Heibei, China

**Keywords:** Antibodies, SARS virus, Virus-host interactions, Viral infection

Correction to: *Nature Communications* 10.1038/s41467-020-15562-9, published online 27 March 2020.

The original version of the Supplementary Information associated with this Article contained inadvertent errors in Supplementary Fig. 2, in which the image showing results for experiments with TMPRSS4 and SARS-CoV S was duplicated for experiments with TMPRSS4 and SARS-CoV-2 S and the image showing results for experiments with TMPRSS11A and SARS-CoV S was duplicated for experiments with TMPRSS11A and SARS-CoV-2 S. The HTML has been updated to include a corrected version of the Supplementary Information.

## Supplementary information

Supplementary information

